# Caries and Necrosis of the Os Humeri, Requiring Amputation of the Arm at the Shoulder Joint

**Published:** 1862-04

**Authors:** Charles Winne


ART. IV.—*Caries and Necrosis of the Os Humeri, requiring Amputation of the Arm at the shoulder joint, by* CHARLES WINNE, M. D.

In the month of May, 1852, a German youth of nineteen years of age was admitted into the Hospital of the Sisters of Charity, Buffalo, for a protracted and painful disease of the left arm; he had been sickly from childhood, and had suffered always, poverty and privation. About one year and a half before the time of his entrance into the Hospital he was exposed to inclement weather, and was seized with pain supposed to be rheumatic, in the left shoulder joint, which became permanent, and gradually extended downward along the shaft of the bone; the pain was very severe and subject to paroxysms of augmentation and decline, but the patient was never free from a dull, deep aching. Gradually enlargement of the bone and swelling of the surrounding tissues advanced. Abscesses formed and were either opened or broke spontaneously in various points from the middle of the arm to the acromion and along the clavicle and in the axilla. His general health and strength failed; he became emaciated and very much debilitated, and in this condition he was placed under my care; his appetite was gone, his complexion cadaverous, his pulse small and feeble, had excessive night sweats and was wearing out with irritative fever. A thorough examination was made to ascertain the condition of the bone by probing it, and the conclusion arrived at from this exploration and his general condition was, that life could only be saved by a removal of the arm at the shoulder joint. He assented readily and cheerfully to submit to the operation, and it was successfully performed a short time after his admission. His recovery was rapid, and fortunately attended with no accidents. His health was entirely re-established by the removal of the irritating cause. His system underwent an entire change. He is now in the employ of one of the Railroad Companies in this City, acting as flagman, and is a large, robust and healthy man.

